# Using Multi-Layer Bidirectional Distillation to Enhance Local and Global Features for Action Recognition

**DOI:** 10.3390/s25226849

**Published:** 2025-11-09

**Authors:** Shilu Kang, Hua Huo, Jiaxin Xu, Aokun Mei, Chen Zhang

**Affiliations:** College of Information Engineering and Artificial Intelligence, Henan University of Science and Technology, Luoyang 471000, China; 185300000021@stu.haust.edu.cn (S.K.); 205400000023@stu.haust.edu.cn (J.X.); 210321050459@stu.haust.edu.cn (A.M.); 250410040094@stu.haust.edu.cn (C.Z.)

**Keywords:** human action recognition, 3D CNN, video Transformer, complementary mechanisms, knowledge distillation, feature fusion

## Abstract

Different action recognition tasks exhibit significant variations in their reliance on local versus global features. Particularly for long-video understanding, dynamically balancing the contributions of both has become a critical challenge for improving recognition accuracy. This paper proposes a Multi-Layer Bidirectional Distillation Model (MBD) based on the two-stream architecture. It employs 3D CNN and video Transformer to capture local and global spatio-temporal features of videos, respectively, aiming to explore the complementary mechanisms between these two feature types and facilitate their synergistic enhancement across diverse recognition task scenarios. The model quantifies feature contributions across specific recognition tasks to map feature dominance, categorizing videos into distinct feature-dominant groups. This mechanism provides a clear direction for knowledge transfer, overcoming the limitations of traditional unidirectional knowledge distillation. Bidirectional knowledge distillation is then performed at the intermediate and final layers, training the model to learn complementary relationships between features and addressing the issue of insufficient representational capacity of non-dominant features. During inference, an adaptive fusion strategy based on feature dominance is adopted, achieving feature fusion via dynamic weighted summation. This mechanism effectively suppresses noise interference from non-dominant features while maximizing the discriminative advantages of dominant features. The MBD model undergoes systematic comparative experiments across four classic action recognition benchmarks (UCF101, HMDB51, Kinectics-400, Something-Something V2). The results demonstrate that the MBD model not only excels in short-video recognition but also outperforms in analyzing complex actions under long-video scenarios.

## 1. Introduction

Human action recognition, as one of the core tasks in computer vision, finds extensive applications in intelligent surveillance, human–computer interaction, sports analysis, and video retrieval. With the advancement of imaging technology and the popularization of shooting equipment, video data has experienced explosive growth, primarily manifested in increased volume, extended duration, and richer content. While massive amounts of high-definition video provide a data foundation for video understanding, they also impose higher demands on recognition technologies. Traditional methods primarily rely on manually designed features (e.g., Histogram of Oriented Gradient (HOG) [1], Spatial–Temporal Interest Points (STIP) [2]) to describe action patterns, which no longer meet current application demands. With the advancement of deep learning technology, various deep learning frameworks have achieved remarkable results in computer vision. An increasing number of researchers are applying these frameworks to action recognition. These approaches can be broadly categorized into two-stream 2D Convolutional Neural Network (CNN)-based methods [3], 3D CNN-based methods [4], Recurrent Neural Network (RNN)-based methods [5], and video Transformer-based methods [6]. Given the powerful representational capabilities and outstanding performance of these deep learning methods, current mainstream research in action recognition focuses on designing different types of deep learning frameworks.

**Figure 1:**
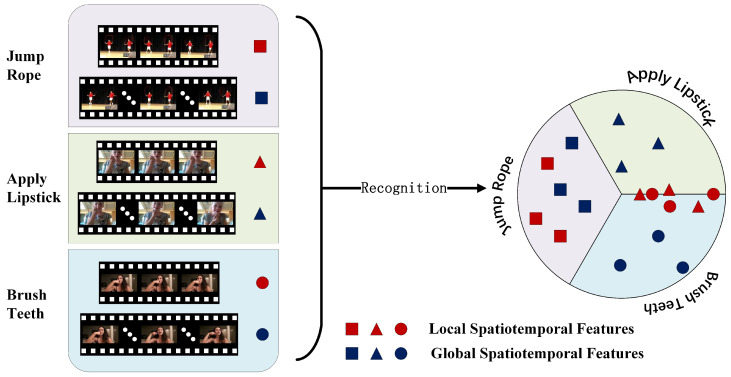
Discriminative contribution of local vs. global spatio-temporal features in classification tasks. (Note: Each category illustration (**left**) contains three representative video samples.)

As shown in Figure 1, repetitive actions rely more heavily on local spatio-temporal features during recognition, such as “jumping rope”, “pull-ups”, and “push-ups”. Non-repetitive actions exhibit stronger dependence on global features. For instance, “rafting” might be misclassified as “rowing” without global features, “pole vaulting” could be identified as “high jumping”, and “brushing teeth” might be mistaken for “applying lipstick”. Figure 1 illustrates two key challenges in action recognition: videos present more complex scenes than images, and multi-feature fusion can enhance the performance of action recognition models; video sequences contain both local spatio-temporal features and global spatio-temporal features, which exhibit varying sensitivities to human actions in videos across different action recognition tasks.Therefore, in an action recognition task, determining which features significantly contribute to recognition and enhancing their influence is crucial. Some features contribute little to recognition or may even degrade performance; reducing their negative impact is a key approach to improving model robustness. This concept aligns with the selective attention mechanism evolved by the nervous system [7,8]: by focusing on key stimuli through eye movements and head turns while suppressing irrelevant information, it optimizes the allocation of limited cognitive resources. This article combines multi-feature fusion with dominant feature selection to establish a biomimetic action recognition model, which not only enhances the model’s recognition accuracy but also ensures the scientific rigor of its construction logic.

Three-dimensional CNNs leverage the spatio-temporal convolution properties of 3D convolutional kernels to effectively capture local spatio-temporal features across the temporal dimension. However, the limited receptive field of CNNs constrains their ability to model long-term spatio-temporal features. To capture global spatio-temporal features of videos, Inflated 3D ConvNet (I3D) [9] inputs entire videos into the model for recognition accuracy testing; Slowfast [10] samples multiple segments from videos to obtain more comprehensive information. Acquiring richer video features by increasing input segments inevitably consumes substantial computational resources. The self-attention mechanism in video Transformers enables interaction between any two frames, overcoming the limitations of local convolutions. Each token learns the attention distribution across the entire sequence, thereby encoding contextual information into the video representation. Consequently, video Transformers inherently possess an advantage in capturing global spatio-temporal features [11]. However, their difficulty in capturing local details limits recognition accuracy. Video Swin Transformers (VideoSwin) [12] introduce local window attention to enhance fine-grained recognition. Particularly in complex scenes, Transformers suffer significant performance degradation due to noise interference. Multi-scale Vision Transformers (MViTs) [13] designed multi-scale attention to suppress background noise. In summary, 3D CNsN and video Transformers each have strengths and weaknesses in action recognition. How to make these two frameworks complementary and mutually reinforcing will be the focus of this research.

Knowledge distillation [14,15,16], originally developed as a model compression technique in deep learning, aims to transfer knowledge from complex “teacher models” to lightweight “student models” for model compression and acceleration. Recent research reveals that knowledge distillation transcends compression, emerging as a key technology for enhancing model representation capabilities and exploiting feature complementarity [17,18,19]. Simply put, it transfers the superior representational capabilities of the “teacher model” to the “student model” through feature alignment, serving as a bridge for complementary relationships between features across heterogeneous models.

This paper constructs the Multi-Layer Bidirectional Distillation Model (MBD model) based on the two-stream architecture. The MBD model leverages the inherent properties of 3D CNNs and video Transformers to extract local spatio-temporal features and global spatio-temporal features from videos, respectively. Feature dominance is mapped by quantifying the probability distribution of the distance between specific feature representations and ground truth labels, thereby categorizing samples into a local feature-dominant group or global feature-dominant group. Building upon this, a multi-layer bidirectional knowledge distillation model is designed. For samples in the local feature-dominant group, a 3D CNN serves as the “teacher model”, while a video Transformer acts as the “student model”. Knowledge distillation occurs at the intermediate and final layers to enhance the video Transformer’s representational capabilities. Similarly, the global feature-dominant group undergoes reverse knowledge distillation to improve the 3D CNN’s representational abilities. Multi-layer bidirectional knowledge distillation transfers rich classification discriminative information from dominant features to non-dominant features through feature alignment, effectively enhancing the representational learning capability of non-dominant features and achieving synergistic optimization of local and global features. Consequently, it improves the accuracy and robustness of video classification. During the feature fusion stage, a dynamic fusion strategy is employed. The weights assigned to local and global spatio-temporal features during fusion are determined based on the probability distribution of distances between feature representations and ground truth labels. This dynamic fusion further exploits complementary enhancement relationships among features, thereby achieving optimal model performance.

## 2. Method

**Figure 2:**
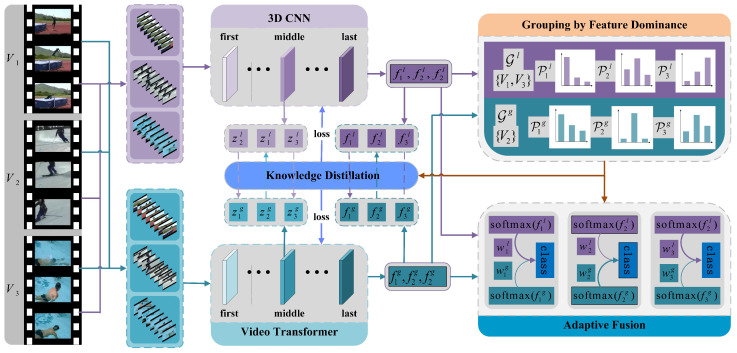
An illustration of the Multi-Layer Bidirectional Distillation Model (MBD model). When capturing local spatio-temporal features, take random sample segments from videos and feed then into 3D CNN for feature extraction. For global spatio-temporal feature, video frames are sampled at fixed intervals and the temporal order is concatenated before being input into the video Transformer for feature extraction. The dominance of features is mapped by quantifying the certainty of probability distributions representing the distance between specific feature representations and ground truth labels, thereby grouping videos. Based on feature dominance, bidirectional knowledge distillation occurs between the model’s intermediate and final layers, transferring rich classification discriminative information from dominant features to non-dominant ones. Finally, a dynamic feature fusion strategy assigns weights to local and global spatio-temporal features during the fusion stage according to their importance, thereby achieving optimal model performance.

As shown in Figure 2, the MBD model proposed in this study comprises two parallel pathways: one employs a 3D CNN to capture the local spatio-temporal feature representation $f_{i}^{l}$ of the video, while the other utilizes a video Transformer to capture the global spatio-temporal feature representation $f_{i}^{g}$. Here, *i* denotes the index variable of the video during the training iteration process. The probability distributions of the distances between these two types of feature representations and the classification labels $I_{k}$ are computed separately. The dominance of different features in the video classification task is measured by the difference in the maximum values of these two probability distributions. Samples where local spatio-temporal features dominate the classification task are categorized into the local feature-dominant group $G^{l}$; conversely, samples where global spatio-temporal features dominate the classification task are assigned to the global feature-dominant group $G^{g}$. Subsequently, multi-layer bidirectional knowledge distillation transfers rich classification discriminative information from dominant features to non-dominant features, thereby enhancing the representational learning capability of non-dominant features. Here, the dominant features act as the “teacher”, while the corresponding non-dominant features serve as the “student”. The “teacher” guides the “student” through optimization, exploiting complementary relationships between different features. The knowledge distillation process occurs not only in the final layer of the neural network but also in its intermediate layers. During the testing phase, the MBD model employs a dynamic feature fusion strategy to integrate local spatio-temporal feature representations with global spatio-temporal feature representations. This strategy prioritizes dominant features, ensuring the accuracy and robustness of classification results.

### 2.1. Backbone Network

Based on the research motivation and the inherent characteristics of neural networks, the two pathways of the MBD model employ distinct backbone networks for feature extraction. CNNs have achieved remarkable success in computer vision. Three-dimensional CNNs efficiently capture local spatio-temporal features in the temporal domain through spatio-temporal joint convolution and local receptive field mechanisms. They can directly model spatio-temporal correlations and are widely applied in tasks such as video classification, action detection, and real-time analysis. This study employs 3D CNNs (e.g., C3D [20], R3D [21], and R(2+1)D [22]) to capture local features within videos. Notably, the MBD model is generic, meaning any 3D CNN can be integrated into the MBD model framework. Randomly sampled video segments with fixed frames are input into the 3D CNN. The video is classified based on the local spatio-temporal features extracted from these segments.

Traditional 3D convolutional networks rely on local convolutional kernels with limited receptive fields, making it difficult to capture long-term temporal dependencies in videos [23,24,25]. Longformer [26] combines sliding-window local context self-attention mechanisms with task-specific global attention mechanisms. By enabling attention to all tokens in the input sequence through global attention, it overcomes the limitations of short-fragment processing. A video Transformer network (VTN) [6] employs an enhanced Longformer as its temporal encoder, naturally inheriting its global modeling advantages. Such Transformer-based models can process sequences spanning thousands of tokens. VTN combines sliding-window attention with global attention to capture long-term dependencies while reducing computational complexity.

**Figure 3:**
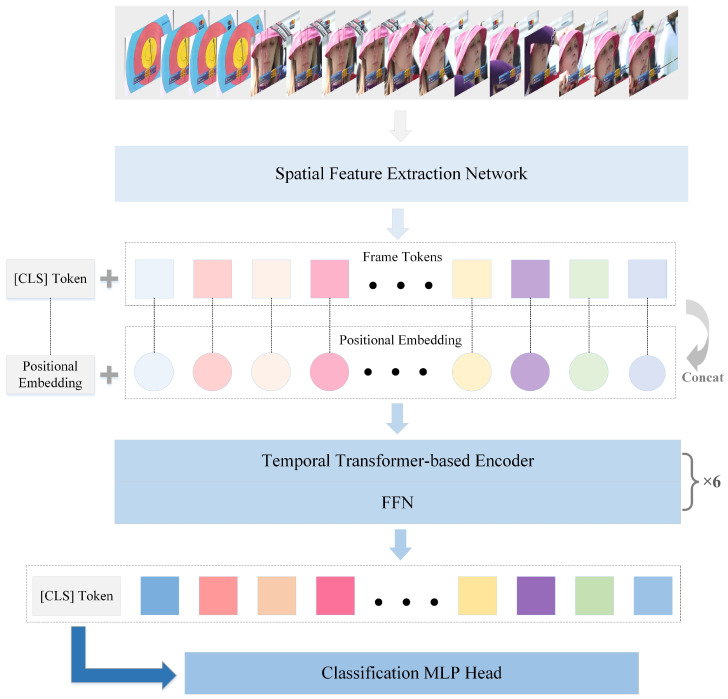
Video Transformer architecture.

Inspired by VTN, the MBD model employs a video Transformer architecture to capture global features in videos. As shown in Figure 3, the video Transformer architecture consists of a spatial feature extraction network and a Longformer-based temporal attention encoder. Uniformly sampling a fixed number of frames from long videos, the spatial feature extraction network extracts spatial semantics frame-by-frame, generating frame-level token sequences. A special classification token [CLS] is added to the front end of each sequence, and a unique position identifier is assigned to each token to encode its temporal order. A 3-layer Longformer temporal encoder with 12 attention heads per layer is used to extract features from the token sequence, producing the final video representation associated with the classification token, which is fed into the designated classification MLP head. The classification MLP head maps this final video representation to the action category space, producing the final classification result.

### 2.2. Dominant Feature Grouping

Different features in distinct videos contribute differently to recognition tasks, with some features even negatively impacting recognition accuracy. Repetitive actions rely more heavily on local spatio-temporal features during recognition, while non-repetitive actions depend more on global spatio-temporal features. Therefore, identifying the features that contribute most significantly to correct classification in specific videos for action recognition tasks is crucial; these are termed dominant features. Dominant features typically contain richer discriminative information and warrant special attention in action recognition tasks. In this paper, samples are grouped based on feature contributions to recognition tasks: samples where local spatio-temporal features dominate classification tasks are assigned to the local feature-dominant group $G^{l}$, while others are assigned to the global feature-dominant group $G^{g}$.

To determine whether the dominant feature of a specific sample is a local or global spatio-temporal feature, this study maps feature dominance by quantifying the certainty of the probability distribution describing the distance between a specific feature representation and the ground truth label $I_{k}$. Specifically, for each sample, the probability distributions of the distances between both its local and global spatio-temporal feature representations and the ground truth label are computed. The maximum value of these probability distributions is then used to measure feature dominance, thereby classifying samples into either the local feature-dominant group $G^{l}$ or the global feature-dominant group $G^{g}$. In practical applications, particularly in deep learning, Softmax normalization is commonly employed to evaluate the probability distribution of distances between feature representations and the ground truth label [27]. The probability distribution of the distance between a specific feature and the ground truth label in a given video during a training iteration can be expressed as follows:

(1) $$P\left({\hat{y}}_{i}=k\right)=\frac{\exp(-D\left(f_{i}^{h},I_{k}\right))}{\sum_{k^{\prime }}^{N}\exp(-D\left(f_{i}^{h},I_{k^{\prime }}\right))}\;h\in \left\{l,g\right\}$$

here $f_{i}^{h}$ denotes the *h* feature representation of the *i*-th sample, where *l* represents local features and *g* denotes global features. $I_{k}$ is the ground truth label of the sample in class *k*. *N* denotes the mini-batch size for each training iteration during the training process. D is the distance metric function, typically employing Euclidean Distance. The dominance of a feature is measured by the maximum element $m_{i}^{h}$ in the probability distribution of distances between the specific feature representation and the ground truth label, indicating the most confident feature classification for *h* feature of sample *i*:

(2) $$m_{i}^{h}=\max_{k\in \{1,\ldots ,N\}}\;P\left({\hat{y}}_{i}=k\right)$$

Then, samples within the mini-batch are grouped based on the maximum element of the probability distribution. If the maximum element $m_{i}^{l}$ of the probability distribution represented for the local spatio-temporal features is greater than the maximum element $m_{i}^{g}$ of the probability distribution represented for the global spatio-temporal features, it indicates that the local spatio-temporal features of this sample contain rich discriminative information during the recognition process. This sample is assigned to the local feature-dominant group $G^{l}$. Conversely, if the global spatio-temporal features of the sample contain rich discriminative information during the recognition process, the sample is assigned to the global feature-dominant group $G^{g}$:

(3) $$G^{l}=\left\{\left(f_{i}^{l},f_{i}^{g}\right)|m_{i}^{l}>m_{i}^{g}\right\}$$

(4) $$G^{g}=\left\{\left(f_{i}^{l},f_{i}^{g}\right)|m_{i}^{l}<m_{i}^{g}\right\}$$

Samples are classified into $G^{l}$ and $G^{g}$ based on the richness of discriminative information contained in specific features. This approach enables the exploration of complementary relationships among different features, mitigates the negative impact of non-dominant features on recognition tasks, and enhances their representational learning capabilities.

### 2.3. Multi-Layer Bidirectional Distillation

This section details the multi-layer bidirectional knowledge distillation strategy that transfers rich classification discriminative information from dominant features to non-dominant features, thereby enhancing the representation learning capability of non-dominant features. Knowledge distillation is a model optimization technique whose core objective is to transfer knowledge from a high-performance, feature-rich “teacher model” to a “student model” with relatively scarce discriminative information. The “teacher model” guides the “student model” to optimize through feature alignment, commonly achieved via KL divergence based on logits values or mean squared error (MSE) based on feature distances.

When using KL divergence as the alignment method, the output feature of the *i*-th sample is $f_{i}$, which generates a probability distribution for soft labels via a softmax function with temperature parameter *T*:

(5) $$p\left(c\right)=\frac{\exp(f_{i,c}/T)}{\sum_{n=1}^{C}\exp(f_{i,c}/T)}$$

*C* denotes the total number of action classes in the dataset, equivalent to the dimensionality of $f_{i}$, where $f_{i,c}$ represents the value of the *c*-th dimension of $f_{i}$. p(c) indicates the distribution probability when soft labels are assigned to class *c*. It is important to note that the probability distributions in Equations (1) and (5) represent different concepts: P(k) denotes the probability distribution of the distance between the feature representation and the ground truth label, while p(c) denotes the probability distribution of the classification prediction for the feature representation. KL divergence measures the “relative entropy” between two probability distributions, serving as an asymmetric metric of their dissimilarity [16,28]:

(6) $$S_{KL}\left(p^{1},p^{2}\right)=\sum_{i=1}^{C}p_{i}^{1}\log\frac{p_{i}^{1}}{p_{i}^{2}}$$

$p^{1}$ and $p^{2}$ denote the probability distributions output by the “teacher model” and “student model”, respectively. Using KL divergence for feature alignment directly reflects the guidance of the “teacher” distribution on the “student”, emphasizing that the category deemed more probable by the “teacher” exerts a greater influence on the “student”.

When using MSE as the alignment metric, the difference between two feature representations is measured by the distance between them:

(7) $$S_{MSE}\left(f^{1},f^{2}\right)=|f^{1}-f^{2}{|}^{2}$$

$f^{1}$ and $f^{2}$ represent the feature representations output by the “teacher model” and “student model”, respectively. The process of feature alignment using MSE is computationally simple but less sensitive to overall differences in distributions. Both alignment methods will be utilized below according to specific requirements.

In this paper, dominant features are regarded as the ”teacher“, and the corresponding non-dominant features act as the ”student“. Knowledge distillation is applied in the intermediate and final layers to transfer discriminative information from the ”teacher“ to the ”student“, thereby enhancing its representational learning capability. Effective knowledge distillation can exploit complementary relationships among different features and mitigate the detrimental effects of noise on recognition tasks. During training iterations, a mini-batch contains *N* samples, with *A* samples in the local feature-dominant group $G^{l}$ and *B* samples in the global feature-dominant group $G^{g}$, where A+B=N. Samples in the local feature-dominant group use local spatio-temporal features as “teachers” to guide the “student” global spatio-temporal feature in learning richer discriminative information. Samples from the global feature-dominant group act as ”teachers“ using global spatio-temporal features, guiding the “students” local spatio-temporal features to learn richer discriminative information.

Bidirectional knowledge distillation is performed at both the intermediate layers and the final layer of the model. Bidirectional knowledge distillation at the intermediate layer focuses on “detail alignment”: intermediate layers extract low-abstraction, high-detail features, such as human joint movement trajectories and preliminary associations of object interactions in scenes. If distillation occurs only at the final layer, the supplementary role of these fine-grained details for non-dominant features would be lost. Bidirectional knowledge distillation at the final layer, in contrast, centers on “semantic alignment”: features at the final layer are highly abstract. Distilling solely at intermediate layers fails to transfer such high-level semantics to non-dominant features, limiting their ability to understand complete temporal patterns and global scene context. Conducting knowledge distillation simultaneously at intermediate and final layers achieves the bidirectional transfer of details and semantics. Through the alignment and complementarity of multi-layer features, this approach maximizes the learning capacity of non-dominant features, ultimately enhancing the model’s performance in complex action recognition tasks.

#### 2.3.1. Perform Bidirectional Knowledge Distillation at the Final Layer

The logit values $f_{i}^{l}\in R^{C}$ and $f_{i}^{g}\in R^{C}$ obtained from the final layer of the 3D CNN and video Transformer represent the local spatio-temporal feature representation and global spatio-temporal feature representation of the *i*-th sample, respectively. Knowledge distillation is performed using the KL divergence minimization method. When the sample belongs to the global feature-dominant group, the loss function is expressed as follows:

(8) $$L_{KL}^{l}=\frac{1}{B}\sum_{\left(f_{i}^{l},f_{i}^{g}\right)\in G^{g}}S_{KL}\left(p_{i}^{g},p_{i}^{l}\right)$$

Training 3D CNNs using $L_{KL}^{l}$ can enhance the representational learning capability of local features. When samples belong to the dominant group of local feature-dominant group, the loss function is obtained as follows:

(9) $$L_{KL}^{g}=\frac{1}{A}\sum_{\left(f_{i}^{l},f_{i}^{g}\right)\in G^{l}}S_{KL}\left(p_{i}^{l},p_{i}^{g}\right)$$

Training video Transformers using $L_{KL}^{g}$ can enhance the representational learning capability of global features.

#### 2.3.2. Perform Bidirectional Knowledge Distillation at the Intermediate Layer

The local spatio-temporal feature representation of the *i*-th sample obtained by the 3D CNN in the intermediate layer is denoted as $z_{i}^{l}\in R^{T^{l}}$. The global spatio-temporal feature representation of the *i*-th sample obtained by the video Transformer in the intermediate layer is denoted as $z_{i}^{g}\in R^{T^{g}}$. $T^{l}$ and $T^{g}$ denote the dimensions of the local spatio-temporal feature representation and the global spatio-temporal feature representation, respectively. Due to differing feature dimensions at the intermediate layer, a projection function ϕ is required for dimension alignment. $\phi^{g}$ denotes the transformation of the local spatio-temporal feature representation $z_{i}^{l}$ to the dimension of the global spatio-temporal feature representation $z_{i}^{g}$, while $\phi^{l}$ denotes the transformation from the dimension of the global spatio-temporal feature representation $z_{i}^{g}$ to that of the local spatio-temporal feature representation $z_{i}^{l}$. Knowledge distillation is performed by minimizing the MSE. When samples belong to the globall feature-dominant group, the loss function is

(10) $$L_{MSE}^{l}=\frac{1}{B}\sum_{\left(f_{i}^{l},f_{i}^{g}\right)\in G^{g}}S_{MSE}\left(z_{i}^{g},\phi^{g}\left(z_{i}^{l}\right)\right)$$

Training 3D CNNs using $L_{MSE}^{l}$ can enhance the representational learning capability of local features. When samples belong to the dominant group of local feature-dominant group, the loss function is obtained as follows:

(11) $$L_{MSE}^{g}=\frac{1}{A}\sum_{\left(f_{i}^{l},f_{i}^{g}\right)\in G^{l}}S_{MSE}\left(z_{i}^{l},\phi^{l}\left(z_{i}^{g}\right)\right)$$

Training video Transformers using $L_{MSE}^{g}$ can enhance the representational learning capability of global features.

### 2.4. Composite Loss Function

The two backbone networks in the MBD model—the 3D CNN and the video Transformer—learn distinct features. Based on the preceding description, different loss functions are constructed to optimize each pathway. The loss function for the 3D CNN, which extracts local spatio-temporal features, can be defined as follows:

(12) $$L^{l}=L_{CN}^{l}+\alpha^{l}L_{KL}^{l}+\beta^{l}L_{MSE}^{l}$$

The loss function for the video Transformer, which extracts global spatio-temporal features, can be defined as

(13) $$L^{g}=L_{CN}^{g}+\alpha^{g}L_{KL}^{g}+\beta^{g}L_{MSE}^{g}$$

$L_{CN}^{l}$ and $L_{CN}^{g}$ denote the cross-entropy loss functions for the 3D CNN and video Transformer, respectively, ensuring the model’s fundamental representational capability. When a sample belongs to the global feature-dominant group, $\alpha^{l}$ denotes the weight of the loss $L_{KL}^{l}$ obtained from knowledge distillation at the final layer, while $\beta^{l}$ denotes the weight of the loss $L_{MSE}^{l}$ obtained from knowledge distillation at the intermediate layer. Similarly, $\alpha^{g}$ and $\beta^{g}$ denote the weights for losses $L_{KL}^{g}$ and $L_{MSE}^{g}$, respectively. The MBD model optimized using $L^{l}$ and $L^{g}$ not only accurately represents the scenes depicted in the video but also excavates potential complementary relationships among features, thereby enhancing the accuracy of recognition tasks.

### 2.5. Adaptive Fusion Strategy

Multimodal fusion strategies have long posed a challenge for researchers, as simple fusion approaches fail to reflect feature contributions during the fusion stage. This study employs an adaptive feature fusion strategy, where the prediction score for the *i*th sample can be expressed as

(14) $$Prediction\;Score_{i}=w_{i}^{l}soft\max\left(f_{i}^{l}\right)+w_{i}^{g}soft\max\left(f_{i}^{g}\right)$$

$w_{i}^{l}$ and $w_{i}^{g}$ represent the adaptive fusion weights for the local spatio-temporal feature representation and the global spatio-temporal feature representation, respectively. To reflect the importance of specific feature representations in the fusion classification, the maximum element $m_{i}^{l}$ of the probability distribution of the distance between the local spatio-temporal feature representation $f_{i}^{l}$ and the classification label $I_{k}$ is utilized. The maximum element $m_{i}^{g}$ of the probability distribution of the distance between the global spatio-temporal feature representation $f_{i}^{g}$ and the classification label $I_{k}$ are used to determine the adaptive fusion weights:

(15) $$w_{i}^{l}=\frac{m_{i}^{l}}{m_{i}^{l}+m_{i}^{g}},\;w_{i}^{g}=\frac{m_{i}^{g}}{m_{i}^{l}+m_{i}^{g}}$$

## 3. Experimental Setup

This section details the experimental setup for the MBD model. We use Kinetics-400 [9] as the benchmark dataset to pre-train thte 3D CNN and video Transformer. As a large-scale action recognition dataset, Kinetics-400’s diverse actions and spatio-temporal complexity align precisely with the model’s design goal of achieving a “local-global balance”. Abundant samples provide a foundation for training the model to learn complementary relationships between features, while diverse action categories enhance the model’s generalization capability. After pre-training on Kinetics-400, the model underwent end-to-end fine-tuning on the training sets of UCF101 [29], HMDB51 [30], Kinetics-400, and Something-Something V2 (SSV2) [31]. Finally, the action recognition accuracy of the MBD model was evaluated on the test sets of these datasets. All pre-training, fine-tuning, and testing were performed on a GPU cluster using the PyTorch 2.8.0 framework.

### 3.1. Pre-Training 3D CNN

The 3D CNN is pre-trained on the Kinetics-400 dataset. During the training phase, model parameter learning is performed on the training set, and the validation set is used to evaluate the model’s generalization ability. For training, segments are randomly sampled from each video, with each segment containing *L* consecutive video frames. To validate the impact of different segment lengths, models are trained using 16-frame and 32-frame segments in comparative experiments. Video frames within segments are resized to 128×171 pixels, then randomly cropped to generate 112×112 pixel frame sequences. Random horizontal flipping is applied to the entire segment. The training process adopts mini-batch gradient descent with the following configurations: batch size set to 64 and stochastic gradient descent (SGD) as the optimizer. Weight decay is applied to suppress overfitting, with a decay coefficient of 0.0001; momentum is set to 0.9 to accelerate gradient convergence and mitigate local minima issues. The initial learning rate is 0.01 and dynamically decayed according to a cosine curve using the Cosine Annealing Scheduler [32]. The total number of training epochs is set to 45. During the testing phase, video-level Top-1 accuracy is used to evaluate the pre-training effectiveness. Ten segments are uniformly sampled from a single video along its temporal axis. The shorter side of each segment is resized to 128 pixels. Center cropping is applied to each segment to generate a 112×112 region. The final video-level prediction result is obtained by averaging the predictions of these 10 clips.

### 3.2. Pre-Training Video Transformer

The standard ViT-B serves as spatial feature extraction backbone for the video Transformer, which has been pre-trained on the ImageNet dataset. The Longformer and MLP classification head are randomly initialized using a normal distribution with a mean of 0 and a standard deviation of 0.02. Subsequently, end-to-end pre-training is conducted using video segments sampled from the Kinetics-400 dataset. During sampling, the starting frame of each segment is randomly selected. The sampling stride τ is fixed at 8 frames to enhance temporal variability between frames. The sampled video frames are concatenated in temporal order to form continuous video segment. Similar to Section 3.1, the number of video frames *L* in each segment was set to 16 frames and 32 frames, respectively, depending on the specific experimental requirements.

During the data preprocessing stage, all frames in the video clips are first randomly resized, adjusting the shorter side to the range of [256, 320] pixels. Subsequently, uniform random cropping is applied to generate frame sequences of size 224×224 pixels. Finally, random horizontal flipping is performed on the entire clip. During the pre-training of the Video Transformer, the batch size is set to 64, and the optimizer is stochastic gradient descent (SGD). The weight decay coefficient is set to 0.0001, and momentum is set to 0.9. The initial learning rate is set to 0.01, with a cosine annealing learning rate scheduler employed for dynamic decay to ensure sufficient model convergence. The total number of training epochs is set to 60.

During the testing phase, uniformly select 10 starting frames along the video timeline. After each starting frame, sample segments at fixed 8-frame intervals. Each image within a segment is scaled to 256 pixels on its shorter side. A central crop of each segment generates a 224×224 pixel region. The prediction results from these 10 segments are averaged to produce the final video-level prediction.

### 3.3. Cooperative Optimization of Two Pathways

To enable the model to learn the complementary relationship between local spatio-temporal features and global spatio-temporal features during action classification, thereby comprehensively improving action recognition accuracy, a two-stream architecture is constructed by combining a pre-trained 3D CNN with a video Transformer. This architecture simultaneously captures both local and global spatio-temporal features in videos. The dominant feature grouping and multi-layer bidirectional knowledge distillation strategy introduced in Section 2 are employed to learn the complementary relationships among different features.

To evaluate the model’s performance and generalization capability, the MBD model was fine-tuned on different datasets. Given the distinct characteristics of 3D CNNs and video Transformers, different sampling strategies and data preprocessing methods are adopted (as described in Section 3.1 and Section 3.2). Notably, the sampling stride introduced in Section 3.2 is specifically tailored for each dataset. The training process similarly employed mini-batch gradient descent with the following configurations: batch size set to 32; stochastic gradient descent selected as the optimizer; weight decay coefficient set to 0.0001; momentum set to 0.9; and learning rate initialized at 0.005 and decayed using a cosine annealing scheduler. The total number of training epochs was 120.

### 3.4. Inference

To ensure fairness in model comparisons, this paper strictly adheres to evaluation conventions in the video understanding domain by employing three-crop testing [10,24,33]. Three-crop testing involves uniformly sampling 10 segments along the video timeline. These segments are input into a 3D CNN, with the short side scaled to 128 pixels. From each segment, a 112×112 region is cropped from the left, middle, and right positions. Ultimately, each video generates 30 viewpoints for the 3D CNN. The average of the softmax scores across these 30 viewpoints serves as the final local spatio-temporal feature representation for subsequent adaptive fusion. For segments input to the video Transformer, the shorter side is scaled to 256 pixels, and then a 224×224 region is extracted from each of the left, center, and right positions. Ultimately, 30 viewpoints are generated for each video for the video Transformer. Similarly, the average of the softmax scores across the 30 viewpoints is computed as the final global spatio-temporal feature representation for the final adaptive fusion.

Following the adaptive fusion strategy introduced in Section 2.5, the adaptive fusion weights $w_{i}^{l}$ and $w_{i}^{g}$ are computed for the local spatio-temporal feature representation and the global spatio-temporal feature representation, respectively. The final video classification prediction is then obtained by performing a weighted sum of the local spatio-temporal features and the global spatio-temporal features.

## 4. Experiment

This section conducts systematic experimental analysis focused on the performance validation of the MBD model. First, through ablation experiments targeting the model’s core modules (e.g., bidirectional distillation at intermediate layers and bidirectional distillation at the final layer), we incrementally verify the contribution of each component to the overall performance, thereby systematically demonstrating the effectiveness and necessity of the model design. Building upon this foundation, we further explore the model’s optimal configuration to identify its best operating conditions.

To comprehensively evaluate the model’s generalization ability and competitiveness, experiments were conducted on four mainstream action recognition datasets: UCF101, HMDB51, Kinetics-400, and Something-Something V2. The Top-1 and Top-5 action recognition accuracy of the MBD model on these datasets was compared with the publicly available results of state-of-the-art methods to empirically demonstrate the model’s performance advantages and cross-dataset adaptability.

Specifically, for the UCF101 and HMDB51 datasets, the test sets were divided into three splits. The experiments uniformly adopted the average accuracy (Top-1 and Top-5 metrics) across the three test splits as the comparison benchmark to eliminate potential evaluation biases from single-subset divisions. For the Kinetics-400 and Something-Something V2 datasets, which did not adopt a partitioning strategy and provided a unified test set, the model’s Top-1 and Top-5 recognition accuracy on the entire test set was directly reported to ensure the evaluation criteria aligned with the dataset characteristics. Through this multi-dimensional, multi-dataset experimental design, the model’s effectiveness, robustness, and generalization capability are comprehensively validated.

### 4.1. Ablation Experiment

#### 4.1.1. Impact of MBD Model Components on Recognition Performance

To systematically investigate the contribution mechanisms of the model’s core components to action recognition performance, this experiment focuses on three key components: bidirectional knowledge distillation at the intermediate layer, bidirectional knowledge distillation at the final layer, and adaptive fusion strategies. Comparative analyses are conducted on the UCF101 and Kinetics-400 datasets. It is particularly important to note that the dominant feature grouping strategy serves as the foundational prerequisite for implementing these three components. Based on the importance of discriminative information in local spatio-temporal features versus global spatio-temporal features, sample videos are categorized into the local feature-dominant group and global feature-dominant group, providing a structural foundation for subsequent knowledge transfer and fusion. The experiments first established a baseline two-stream architecture for comparison: this architecture comprises a 3D CNN and a video Transformer network, without integrating any knowledge distillation or adaptive fusion modules. As shown in Table 1, on the UCF101 dataset, the baseline model achieved Top-1 and Top-5 recognition accuracies of 95.4% and 99.1%, respectively, while on the Kinetics-400 dataset, the corresponding accuracies are 80.9% and 95.1%. After fully integrating three modules—bidirectional knowledge distillation at the intermediate layer, bidirectional knowledge distillation at the final layer, and an adaptive fusion strategy—into the two-stream architecture, the proposed MBD model was formed. Experimental results demonstrate that the MBD model achieves a 2.0% improvement in Top-1 accuracy (to 97.4%) and a 0.4% improvement in Top-5 accuracy (to 99.5%) on the UCF101 dataset. On the Kinetics-400 dataset, Top-1 and Top-5 accuracies increase by 2.6% (to 83.5%) and 1.3% (to 96.4%), respectively. This significant improvement demonstrates that the MBD model effectively captures the complementary relationship between local spatio-temporal features and global spatio-temporal features through the synergistic interaction of bidirectional knowledge distillation and adaptive fusion, significantly enhancing the model’s representational capabilities.

To further elucidate the independent contributions and interaction mechanisms of each component, ablation experiments were conducted. When integrating only the bidirectional knowledge distillation module at the intermediate layer, the Top-1 accuracy of UCF101 and Kinetics-400 improved by 0.6% (to 96.0%) and 0.8% (to 81.7%), respectively. When integrating only the bidirectional knowledge distillation module at the final layer, the Top-1 accuracy of UCF101 and Kinetics-400 improved by 1.5% (to 96.9%) and 1.6% (to 82.5%), respectively. This demonstrates that the intermediate layer bidirectional knowledge distillation module, constrained by the lower abstraction level of intermediate layer features, yields weaker improvements compared to the final layer module. Features from the final layer contain richer information, enabling dominant features to transfer more discriminative information to non-dominant features. When integrating only the adaptive fusion module, the Top-1 accuracy of UCF101 and Kinetics-400 improved by merely 0.1% (to 95.5%) and 0.2% (to 81.1%), respectively, demonstrating limited effectiveness. This phenomenon indicates that relying solely on feature contribution for dynamic fusion, without the feature complementarity provided by knowledge distillation, struggles to directly enhance model performance. When all three modules are integrated, recognition accuracy reaches its peak on both datasets. This indicates that the dynamic weights assigned to different features facilitate optimal fusion of complementary features, effectively optimizing the model’s ability to learn action class boundaries.

In summary, the experimental results systematically validate the rationality of the MBD model design from two dimensions: independent contributions of components and synergistic effects. By enhancing discriminative information through bidirectional knowledge distillation between the intermediate layer and the final layer, and integrating this with the dynamic regulation of the adaptive fusion strategy, the MBD model ultimately achieves deep complementarity between local and global spatio-temporal features, significantly boosting the accuracy and robustness of action recognition.

#### 4.1.2. Impact of Network Configuration on MBD Model Performance

The two-stream architecture adopted by the MBD model is generic, allowing each branch to select different base configurations. This section conducts comparative experiments focusing on the base network configurations of the local spatio-temporal feature branch (3D CNN) and the global spatio-temporal feature branch (video Transformer), systematically investigating the patterns of influence that different network configurations exert on model parameter counts and recognition performance. As introduced in Section 2.1, the local spatio-temporal feature branch is based on 3D CNNs. R(2+1)D [22] demonstrates superior performance in action recognition tasks compared to traditional 3D CNNs due to its advantages in spatio-temporal feature decomposition. Based on this, the MBD model adopts R(2+1)D as the backbone network for local spatio-temporal feature extraction. R(2+1)D networks are typically extended from ResNet’s residual block architecture. To clarify the impact of depth on performance, experiments compare two typical configurations: R(2+1)D-34 employs the same number of residual blocks (34 layers) as ResNet-34, corresponding to a shallow network structure; R(2+1)D-50 uses the same number of residual blocks (50 layers) as ResNet-50, corresponding to a deep network structure. The global spatio-temporal feature branch adopts a video Transformer architecture (as described in Section 2.1), whose core consists of a 2D spatial feature extraction network and a temporal attention encoder based on Longformer [26]. VTN [6] indicates that varying the number of layers in Longformer has minimal impact on performance. This experiment uniformly adopts a three-layer Longformer architecture to balance performance and efficiency. For the 2D spatial feature extraction network, experiments compared three mainstream options: ViT-B, ResNet101, and ResNet50.

**Figure 4:**
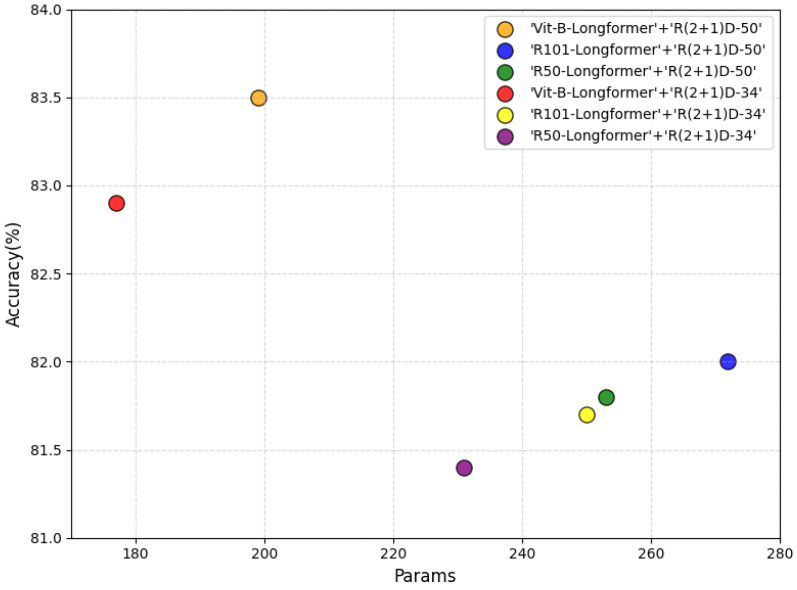
Impact of network configuration on MBD model performance.

Figure 4 illustrates the model parameter count versus Top-1 recognition accuracy on the Kinetics-400 dataset across different network configurations. It is evident that the deeper network architecture R(2+1)D-50 demonstrates superior performance, indicating that deeper R(2+1)D networks enhance the fitting of local spatio-temporal features through the stacking of more complex residual blocks. The choice of the spatial feature extraction network for the video Transformer significantly impacts performance. The ViT-B model achieves the best recognition results for spatial feature extraction, primarily because the Transformer’s self-attention mechanism more efficiently captures global spatial dependencies (such as interactions between different objects). Furthermore, the attention mechanisms of ViT-B and Longformer exhibit inherent synergy in spatio-temporal modeling. Additionally, the MBD model using ViT-B as the spatial feature extraction network requires fewer parameters than other configurations. Therefore, unless otherwise specified, subsequent experiments employ the R(2+1)-50 network for the local spatio-temporal feature branch and the ViT-B-Longformer architecture for the global spatio-temporal feature branch. According to our observations, the FLOPs for the local spatio-temporal feature branch are 10.6 G; the FLOPs for the global spatio-temporal feature branch are 6.1 G.

#### 4.1.3. Performance Analysis of Different Knowledge Distillation Strategies

The primary contribution of this study lies in bidirectional knowledge distillation between the dominant feature group and non-dominant feature group across the model’s intermediate and final layers. This approach meticulously distinguishes dominant and non-dominant feature groups, transferring rich classification discriminative information from dominant features to non-dominant features through knowledge distillation. This breakthrough overcomes the information transfer limitations inherent in traditional unidirectional knowledge distillation. Identifying the appropriate “teacher model” is crucial in knowledge distillation, as it enables the transfer of beneficial information for recognition tasks, thereby enhancing the model’s overall recognition capability. To validate the scientific validity of the proposed dominant and non-dominant feature group classification and the effectiveness of bidirectional knowledge distillation, this section focuses on the core elements of knowledge distillation—the selection of the “teacher model”and hierarchical configuration—and designs multi-dimensional comparative experiments. Experiments are conducted on the Kinetics-400 dataset, with Top-1 recognition accuracy as the evaluation metric. By controlling the sources of the “teacher model” (local spatio-temporal features/global spatio-temporal features/dominant group) and distillation levels (intermediate layer/final layer/combination of both), we thoroughly investigate how different configurations influence model performance.

**Figure 5:**
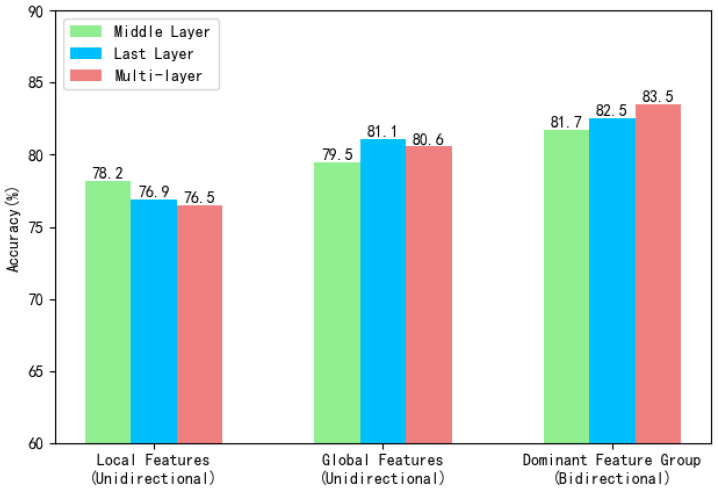
Performance of different knowledge distillation strategies.

As shown in Figure 5, knowledge distillation is performed at the intermediate layer, final layer, and multi-layer using local features, global features, and the dominant feature group as the “teacher model”, respectively. Knowledge distillation using either local or global single features as the “teacher model” is unidirectional, while knowledge distillation using dominant feature groups as the “teacher model” is bidirectional. As shown in the bar chart, unidirectional knowledge distillation using a single feature as the “teacher model” consistently yields weaker results than bidirectional knowledge distillation using the dominant feature group as the “teacher model”. Further analysis reveals the following: In unidirectional experiments, recognition accuracy progressively decreases when using local spatio-temporal features as the “teacher model” across different layers, with all results falling below the accuracy achieved without knowledge distillation (80.9%; see Table 1). This phenomenon can be explained as follows: Many videos in the Kinetics-400 dataset are dominated by global spatio-temporal features. However, transferring discriminative information from the local spatio-temporal feature branch fails to enhance the representational capability of the global spatio-temporal feature branch. Instead, it may introduce noise, reducing the modeling capability of the global spatio-temporal feature branch and consequently lowering overall recognition accuracy. When using global spatio-temporal features as the “teacher model”, recognition accuracy achieved 81.1% by performing knowledge distillation only in the final layer is slightly higher than 80.9%. This phenomenon confirms that the Kinetics-400 dataset contains a higher proportion of videos dominated by global spatio-temporal features. It also indicates that intermediate layers possess lower abstraction levels and weaker representational capabilities for video features, whereas higher layers exhibit stronger representational capabilities as they fuse richer global contextual information. Bidirectional knowledge distillation using the dominant feature group as the “teacher model” achieved optimal performance across all experimental conditions. This demonstrates that the division of dominant feature groups aligns with practical task requirements, ensuring the effectiveness of transferred information. Bidirectional knowledge distillation effectively captures the complementary relationship between local details and global semantics. Local features supplement the global branch’s perception of action details, while global features enhance the local branch’s understanding of the action’s overall context. This synergy significantly boosts the model’s representational capabilities.

#### 4.1.4. The Impact of Segment Length and Knowledge Distillation Alignment Methods

This section conducts systematic experimental analysis centered on two key elements: input segment length and the final layer knowledge distillation alignment strategy. The aim is to clarify their impact patterns on model performance, providing optimal decision-making basis for model configuration. The length of video input segments directly influences the richness of spatio-temporal information captured by the model. Experiments use the number of video frames as a quantitative metric for segment length: 8×8 indicates training and testing with 8-frame segments, while 16×16 denotes training and testing with 16-frame segments, yielding two MBA models with distinct parameter sets. As shown in Table 2, the model trained and tested with 8-frame segments achieves a Top-1 recognition accuracy of 80.3% and a Top-5 accuracy of 95.2% on the Kinetics-400 dataset. These results are significantly lower than those achieved with 16-frame segments. This substantial gap indicates that longer input segments provide the model with more comprehensive temporal contextual information. Balancing computational resources and model efficiency, this article ultimately selects 16-frame segments as the training input. During inference, to further enhance model robustness, each video undergoes three cropping tests using 10 sampled 16-frame segments.

The core objective of knowledge distillation is to transfer discriminative information from the “teacher model” to the “student model”, and the choice of alignment strategy directly impacts the efficiency of knowledge transfer. Common alignment methods in knowledge distillation include KL divergence based on logit values and MSE based on feature distances. During bidirectional knowledge distillation at intermediate layers, MSE is employed for feature alignment due to the high dimensionality of intermediate layer features. For final layer bidirectional knowledge distillation, both alignment methods are viable. To achieve optimal recognition performance, comparative experiments were conducted on Kinetics-400 using both alignment approaches. As shown in Table 3, the Top-5 recognition accuracy differences between the two methods are negligible. However, KL divergence alignment yields a 1.1% higher Top-1 recognition accuracy than MSE alignment. This phenomenon can be attributed to the fact that KL divergence, through the alignment of probability distributions, more directly captures the differences between the “teacher model” and “student model” along the category decision boundary. Fine-grained action recognition tasks exhibit extreme sensitivity to classification boundaries. The KL divergence alignment method enables more precise optimization of the “student model’s” classification decision boundary, thereby enhancing Top-1 accuracy.

#### 4.1.5. Performance Comparison of Different Late Fusion Strategies

Performance optimization of two-stream architectures heavily relies on the design of late fusion strategies [34,35,36,37]. To validate the effectiveness and adaptability of the proposed adaptive fusion strategy, the experimental system evaluated multiple late fusion approaches for integrating local spatio-temporal features with global spatio-temporal features: (1) Average Fusion; (2) Weighted Fusion; (3) Max fusion; (4) Concatenation fusion; (5) Support Vector Machine (SVM); (6) 3D convolution fusion (3D Conv); (7) Bilinear fusion; (8) adaptive fusion strategy.

**Figure 6:**
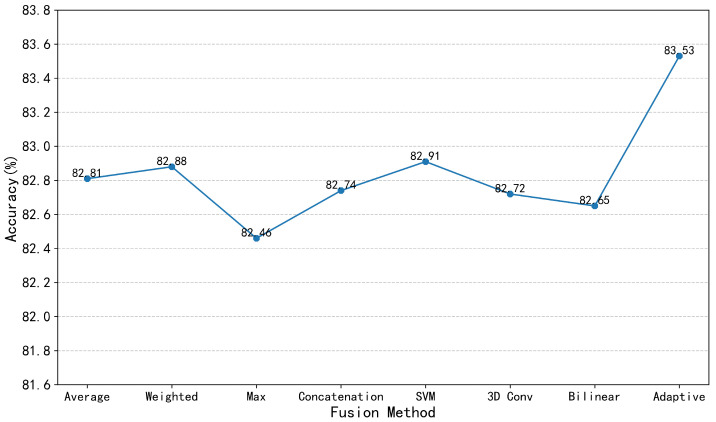
Performance comparison of different late fusion strategies.

The experimental results are shown in Figure 6. On the Kinetics-400 dataset, the Top-1 recognition accuracy of the first seven late fusion strategies cluster within the range of 82.46% to 82.91%, exhibiting minimal performance variation and none surpassing the 83% threshold. The adaptive fusion strategy significantly outperforms other methods with a Top-1 accuracy of 83.53%, validating its effectiveness as the optimal fusion solution for two-stream architectures. The adaptive fusion strategy dynamically adjusts the weighting of local versus global features in fusion by quantifying the certainty of feature representations relative to the ground truth label probability distribution. For action categories reliant on local details, it automatically increases the fusion weight of local features; for those dependent on global temporal patterns, it enhances the contribution of global features. This “on-demand allocation” fusion mechanism effectively leverages the discriminative strengths of different features for specific action categories. The adaptive fusion strategy fundamentally achieves end-to-end optimization of “feature selection-fusion,” thereby reducing noise interference in classification decisions. Experimental results directly validate the rationality of the proposed “dynamic fusion based on feature dominance quantification” approach, providing robust theoretical support for MBD models to fully exploit complementary relationships among features.

#### 4.1.6. Weight Sensitivity Analysis of Composite Loss Functions

Section 2.4 constructs composite loss functions, where the loss for each pathway of the two-stream architecture is composed of a weighted sum of cross-entropy loss, KL divergence loss, and MSE loss. As shown in Equations (12) and (13), within the composite loss function of the 3D CNN, $\alpha^{l}$ and $\beta^{l}$ represent the weights for the KL divergence loss and MSE loss, respectively. In the composite loss function of the video Transformer, $\alpha^{g}$ and $\beta^{g}$ represent the weights for the KL divergence loss and MSE loss, respectively.

**Figure 7:**
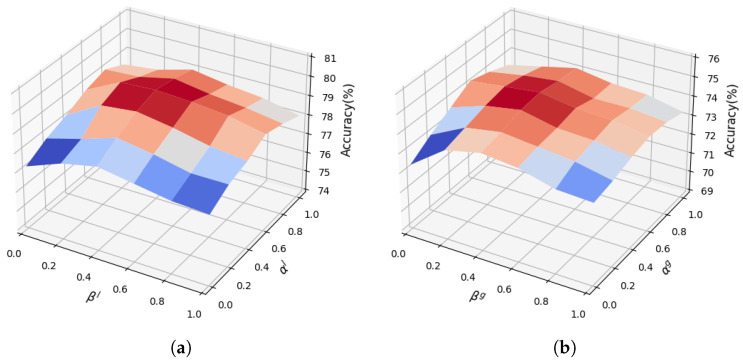
Three-dimensional surface plot of joint effects of weights in loss function. (**a**) The Influence of loss function weights in 3D CNNs; (**b**) the influence of loss function weights in video Transformers.

To determine the optimal weight combination, comparative experiments were conducted using different weight combinations for each branch of the dual-stream architecture. The weights for the KL divergence loss ($\alpha^{l}$ and $\alpha^{g}$) were varied within the range {0,0.2,0.4,0.6,0.8,1.0}, while the weights for the MSE loss ($\beta^{l}$ and $\beta^{g}$) were similarly varied within the range {0,0.2,0.4,0.6,0.8,1.0}. By permuting these weight values, we evaluated the Top-1 recognition accuracy of different pathways under each configuration on the Kinetics-400 dataset. This yielded the 3D surface plot of the combined weight effects in the composite loss function shown in Figure 7. Experimental results reveal that the performance peaks of both branches in the two-stream architecture occur at the same coordinate position [0.6,0.4]. When $\alpha^{l}\alpha^{g}=0.6$ and $\beta^{l}\beta^{g}=0.4$, the highest recognition accuracy achieved by testing the 3D CNN alone was 74.5%, while the highest accuracy achieved by testing the video Transformer alone was 79.6%. The experimental results indicate that when the KL divergence loss weight is 0.6 and the MSE loss weight is 0.4, the recognition accuracy of both pathways in the two-stream architecture simultaneously reaches its maximum. Based on these findings, subsequent experiments in this article uniformly set the KL divergence loss weight to 0.6 and the MSE loss weight to 0.4 for the MBD model. This configuration maximizes the synergistic effect of multi-task losses, enabling the model to achieve high performance in complex action recognition tasks.

### 4.2. Comparison with Existing State-of-the-Art Methods

This study conducts a systematic comparison with current state-of-the-art methods across four classic action recognition benchmarks (UCF101, HMDB51, Kinectics-400, Something-Something V2). The MBD model adopts a two-stream architecture: the local spatio-temporal feature pathway employs a ResNet-50 backbone-based 3D CNN to extract short-range temporal and spatial details, while the global spatio-temporal feature pathway employs a video Transformer with Longformer as its backbone to capture long-range temporal dependencies. Each branch is pre-trained on Kinetics-400, then combined with dominant feature grouping and multi-layer bidirectional knowledge distillation strategies for fine-tuning on each dataset, enabling the model to adapt to various specific tasks. The input segment comprises 16 video frames. Methods involved in the comparison are categorized into three types based on their backbone networks: CNN-based action recognition, RNN-based action recognition, and Transformer-based action recognition. These approaches employ diverse pre-training methods to achieve optimal performance. Most researchers utilize video frames (RGB) as model input, while some incorporate optical flow (Flow) to better capture action features within videos. Methods using both “RGB” and “Flow” inputs draw inspiration from two-stream architecture concepts for modeling.

Table 4 presents the Top-1 recognition accuracy of each method on the UCF101 and HMDB51 datasets. The MBD model achieves a Top-1 recognition accuracy of 97.4% on UCF101, slightly below the 98% achieved by Two-Stream I3D and the 98.6% achieved by Top-Heavy CapsNets, comparable to the performance of Two-Stream R(2+1)D. For the HMDB51 dataset, the Top-1 recognition accuracy is 76.7%, lower than the 80.7% achieved by Two-Stream I3D and the 78.8% achieved by Two-Stream R(2+1)D. Two-Stream I3D, Top-Heavy CapsNets, and Two-Stream R(2+1)D achieve superior recognition performance primarily because both methods utilize both video frame segments and optical flow as inputs. RGB frames provide appearance details, while optical flow directly encodes motion vectors. Cross-modal fusion provides the model with a more comprehensive representation of action. Simultaneously, to capture global video information, Two-Stream I3D and Two-Stream R(2+1)D do not sample input videos during testing but instead use the entire video as input. Whether using optical flow as input or feeding the entire video into the model, these operations undoubtedly consume substantial computational resources.

Table 5 presents the Top-1 and Top-5 recognition accuracies of various methods on the Kinetics-400 dataset. It is evident that the MBD model outperforms both Two-Stream I3D and Two-Stream R(2+1)D across both metrics, achieving 83.5% Top-1 accuracy and 96.4% Top-5 accuracy, respectively. Table 6 presents the recognition accuracy of various methods on the Something-Something V2 dataset. The MBD model again outperforms all comparison methods with a Top-1 accuracy of 73.9% and a Top-5 accuracy of 95.1%, achieving state-of-the-art performance.

A comprehensive comparison of the experimental results reveals that while the MBD model does not surpass Two-Stream I3D and Two-Stream R(2+1)D—which rely on bimodal inputs and test across entire videos—on simpler datasets (UCF101, HMDB51), it maintains comparable performance with significantly reduced computational cost through local–global feature complementarity, dynamic fusion, and efficient input design. Concerning complex long-video datasets (Kinetics-400, Something-Something V2), the MBD model comprehensively outperforms existing methods through optimized long-range temporal modeling and enhanced feature complementarity mechanisms, validating its superiority in complex action recognition tasks. Under controlled input conditions, the MBD model not only excels in short-video recognition but also demonstrates superiority in analyzing complex actions within long-video scenarios. This indicates that by exploring the complementary relationship between local and global spatio-temporal features, the MBD model can precisely capture video motion details and fully map global contextual information.

## 5. Conclusions

This article proposes a Multi-Layer Bidirectional Distillation Model (MBD) for human action recognition, aiming to provide targeted feature learning strategies for local spatio-temporal features and global spatio-temporal features while systematically exploring complementary enhancement relationships among features. The MBD model comprises two pathways: the local spatio-temporal feature pathway employs a 3D CNN to capture short-range temporal and spatial details, while the global spatio-temporal feature pathway leverages a video Transformer to model long-range temporal dependencies and global contextual information. The model determines feature dominance by quantifying the certainty of probability distributions measuring the distance between specific feature representations and ground truth labels, thereby classifying videos into the local feature-dominant group or global feature-dominant group. This mechanism provides a clear direction for knowledge transfer, overcoming the limitations of traditional unidirectional knowledge distillation. Using the dominant feature pathway as the “teacher model”, knowledge distillation occurs at the intermediate and final layers to enhance the representational capacity of the non-dominant pathway. During inference, an adaptive fusion strategy employs dynamically weighted summation for feature integration. This mechanism effectively suppresses noise interference from non-dominant features while maximizing the discriminative advantages of dominant features. The core innovation of the MBD model lies in its technical closed loop of “feature dominance quantification-multi-layer bidirectional knowledge transfer-dynamic fusion”. This loop explores complementary enhancement relationships among features. This resolves the contradiction in traditional models where long-sequence modeling suffers from “loss of local details” and “global semantic ambiguity”. Experimental results demonstrate that the MBD model not only excels in short-video recognition but also excels at handling complex action analysis in long-video scenarios. Currently, the MBD model is suitable for scenarios with limited behavioral categories or less stringent timeliness requirements, such as dangerous motion detection, intelligent monitoring, and video annotation. Many application scenarios are constrained by limited computational resources, hindering the deployment of the MBD model. Subsequent research will focus on lightweight model design while ensuring recognition accuracy, and reduce its reliance on the number of input frames.

## Figures and Tables

**Table 1:** Impact of MBD model components on recognition performance.

Middle Layer Distillation	Final Layer Distillation	Adaptive Fusion Strategy	UCF101		Kinetics-400	
			**Top-1**	**Top-5**	**Top-1**	**Top-5**
✘	✘	✘	95.4	99.1	80.9	95.1
✔	✘	✘	96.0	99.2	81.7	95.6
✘	✔	✘	96.9	99.4	82.5	96.0
✘	✘	✔	95.5	99.1	81.1	95.4
✔	✔	✘	97.0	99.5	82.9	96.2
✔	✔	✔	97.4	99.5	83.5	96.4

**Table 2:** Impact of varying segment lengths on experimental results.

Clip Length	Kinetics-400	
	**Top-1**	**Top-5**
8×8	80.3	95.2
16×16	83.5	96.4

**Table 3:** Experimental results under different alignment strategies.

Alignment Strategies	Kinetics-400	
	**Top-1**	**Top-5**
KL Divergence	83.5	96.4
MSE	82.4	96.1

**Table 4:** Comparison with state-of-the-art works on UCF101 and HMDB51.

Method	UCF101	HMDB51
	**Top-1**	**Top-1**
Two-stream [3]	88.0	59.4
LRCN [5]	82.7	-
L2STM [38]	93.6	61.3
Two-stream I3D [9]	98.0	80.7
Two-stream R(2+1)D [22]	97.4	78.8
TSN [34]	94.2	69.4
TSM [39]	94.5	-
TEA [40]	96.9	73.3
C2LSTM [41]	92.8	66.2
CACL [42]	82.5	48.8
SVT [43]	93.7	67.2
Omi et al. [44]	95.8	75.0
Top-Heavy CapsNets [45]	98.6	80.4
MBD	97.4	76.7

**Table 5:** Comparison with state-of-the-art works on Kinetics-400.

Method	Backbone	Pre-Training	Input	Kinetics-400	
				**Top-1**	**Top-5**
Two-Stream I3D [9]	InceptionV1	ImageNet+ Kinetics-400	RGB, Flow	74.2	91.3
Two-Stream R(2+1)D [22]	ResNet50	Sports-1M	RGB, Flow	75.4	91.9
NL I3D [24]	ResNet101	ImageNet	RGB	77.7	93.3
TSN [34]	InceptionV3	ImageNet	RGB, Flow	72.5	90.2
TSM [39]	ResNet50	ImageNet	RGB, Flow	74.7	91.4
SlowFast [10]	ResNet50	No	RGB	77.0	92.6
TEA [40]	ResNet50	ImageNet	RGB	76.1	92.5
X3D [25]	ResNet	No	RGB	76.0	92.3
MViT [13]	MViT-B	No	RGB	78.4	93.5
TimeSformer [46]	ViT-B	ImageNet21K	RGB	78.0	93.7
TDN [47]	ResNets50	ImageNet	RGB	78.4	93.6
ViT-B-VTN [6]	Longformer	ImageNet21K	RGB	79.8	94.2
TANet [48]	ResNet101	ImageNet	RGB	78.4	93.5
VideoMAE [49]	ViT-B	No	RGB	81.5	95.1
Video Swin [12]	Swin-B	ImageNet21K	RGB	82.6	95.7
UniFormer-B [50]	UniFormer-B	ImageNet	RGB	82.9	95.4
VideoMamba-M [51]	VideoMamba-M	ImageNet	RGB	82.4	95.7
AMD [52]	ViT-B	No	RGB	82.2	95.3
MBD	ResNets50+ Longformer	Kinetics-400	RGB	83.5	96.4

**Table 6:** Comparison with state-of-the-art works on SSV2.

Method	Backbone	Pre-Training	Input	SSV2	
				**Top-1**	**Top-5**
TSM [39]	ResNet50	ImageNet	RGB, Flow	66.6	91.3
SlowFast [10]	ResNet101	Kinetics-400	RGB	63.1	87.6
TDN [47]	ResNets50	ImageNet	RGB	67.0	90.3
TANet [48]	ResNet50	ImageNet	RGB	66.0	90.1
TimeSformer [46]	ViT-B	ImageNet21K	RGB	62.5	-
MViT [13]	MViT-B	Kinetics-400	RGB	67.7	90.9
VideoMAE [49]	ViT-B	No	RGB	70.8	92.4
Video Swin [12]	Swin-B	Kinetics-400	RGB	69.6	92.7
UniFormer-B [50]	UniFormer-B	ImageNet	RGB	71.2	92.8
VideoMamba-M [51]	VideoMamba-M	ImageNet	RGB	68.3	91.4
AMD [52]	ViT-B	No	RGB	73.3	94.0
MBD	ResNets50+ Longformer	Kinetics-400	RGB	73.9	95.1

## Data Availability

We clarify that our research findings are based on the analysis of publicly available datasets: UCF101: https://www.crcv.ucf.edu/research/data-sets/ucf101/ (accessed on 6 September 2025). HMDB51: https://serre-lab.clps.brown.edu/resource/hMBD-a-large-human-motion-database/ (accessed on 6 September 2025). Kinetics-400: https://deepmind.com/research/open-source/kinetics (accessed on 11 September 2025). Something-Something V2: https://github.com/open-mmlab/mmaction2/tree/main/tools/data/sthv2 (accessed on 20 September 2025).
